# Supplementary material for: The Species Effect: Differential Sphingosine-1-Phosphate Responses in the Bone in Human Versus Mouse
Source: Int J Mol Sci. 2024 May 8;25(10):5118. doi: 10.3390/ijms25105118 (PMC11121697; doi:10.3390/ijms25105118)
Supplement: Supplementary file 1 [file ijms-25-05118-s001.zip › Supplementary Figure legends.pdf]

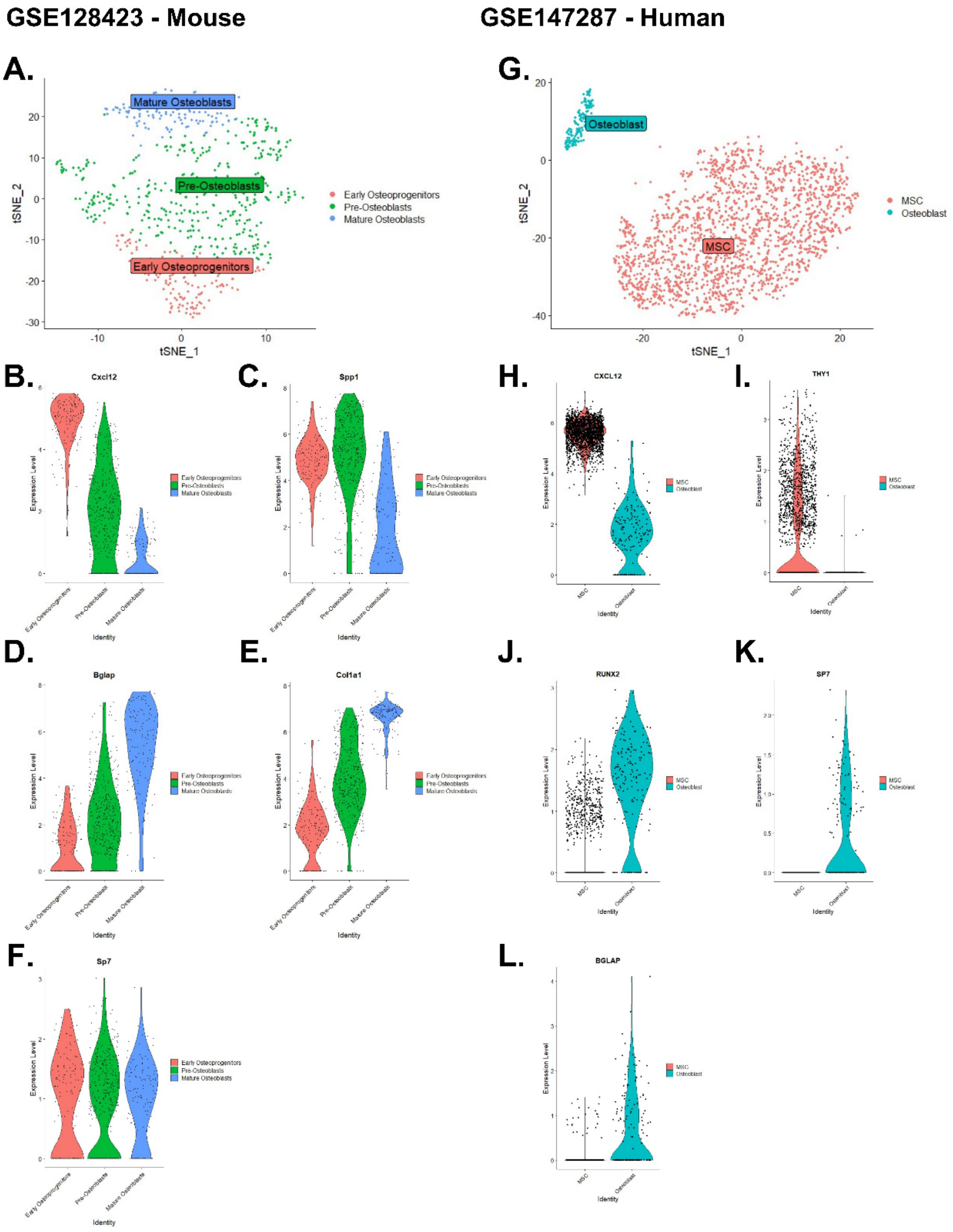

Supplementary Figure S1: Clustering of Osteoblast subsets.

(A-F) Publicly available single cell sequencing dataset GSE128423 (Mouse) was analysed for osteoblasts and subsequently clustered into three subgroups. (**A**) Dotplot of clustered Early Osteoprogenitors, Pre-Osteoblasts and Mature Osteoblasts. (**B-F)** Violin plots showing gene expression of Cxcl12 **(B),** Spp1 (**C**), Bglap (**D**), Col1a1 **(E)** and Sp7 **(F)** in each of the subgroups. **(G-L)** GSE14787 (Human) publicly available dataset was clustered for stromal type cells and further split into mesenchymal stem cells and osteoblasts. (**G**) Dotplot of clustered MSCs and Osteoblasts. **(H-L)** Violin plots showing gene expression of CXCL12 **(H),** THY1 **(I)**, RUNX2 **(J)**, SP7 **(K)** and BGLAP **(L)** in MSC and osteoblast subgroups.

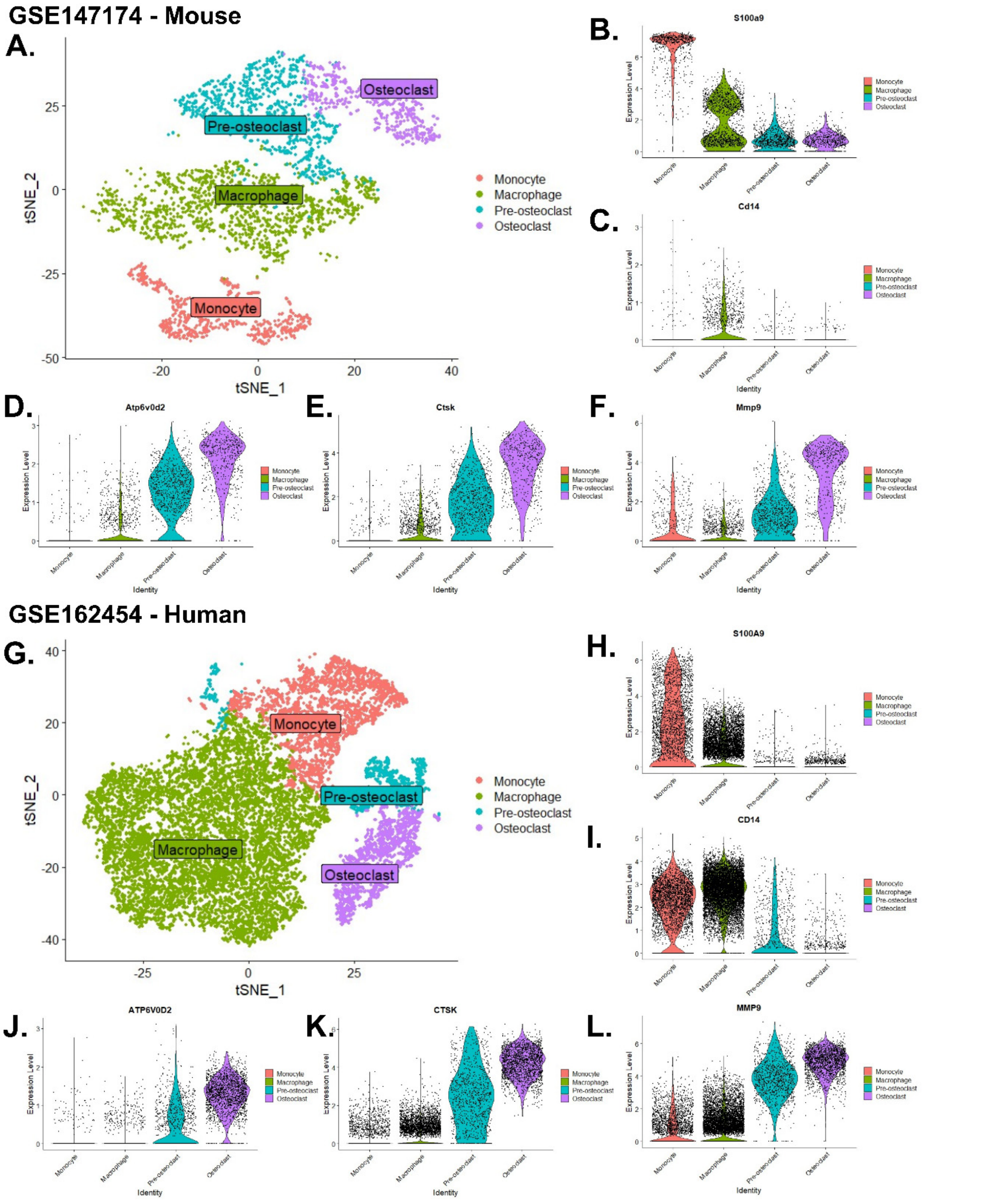

Supplementary Figure S2: Clustering of Osteoclast subsets.
(A-F) Publicly available single cell sequencing dataset GSE147174 (Mouse) was analysed for cells involved in. (**A**) Dotplot of clustered Monocytes, Macrophages. Pre-Osteoclasts and Osteoclasts. (**B-F**) Violin plots showing gene expression of S100a8 **(B),** Cd14 **(C)**, Atp6v0d2 **(D)**, Ctsk **(E)** and Mmp9 **(F)** in each of the subgroups. **(G-L)** The publicly available dataset GSE162454 was clustered for osteoclast lineage cells and clustered into 4 subgroups. (**G**) Dotplot of clustered Monocytes, Macrophages. Pre-Osteoclasts and Osteoclasts. **(H-L)** Violin plots showing gene expression of S100A8 **(H),** CD14 **(I)**, ATP6V0D2 **(J)**, CTSK **(K)** and MMP9 **(L)** in each cell subset.
